# Copper-Chelated Hyperbranched Polyethyleneimines with Antifungal Activity against quiescent conidia and germlings of the opportunistic fungal pathogen Aspergillus nidulans

**DOI:** 10.21203/rs.3.rs-5895555/v1

**Published:** 2025-05-30

**Authors:** Dimitris Tsiourvas, Zili Sideratou, Eleni Mavrogonatou, Dimitris Kletsas, Vicky Sophianopoulou, Spiros Gerostathis

**Affiliations:** Institute of Nanoscience and Nanotechnology, National Centre for Scientific Research “Demokritos”; Institute of Nanoscience and Nanotechnology, National Centre for Scientific Research “Demokritos”; Laboratory of Cell Proliferation and Ageing, Institute of Biosciences and Applications (IBA), National Centre for Scientific Research “Demokritos”; Laboratory of Cell Proliferation and Ageing, Institute of Biosciences and Applications (IBA), National Centre for Scientific Research “Demokritos”; Microbial Molecular Genetics Laboratory, Institute of Biosciences and Applications (IBA), National Centre for Scientific Research “Demokritos”; Microbial Molecular Genetics Laboratory, Institute of Biosciences and Applications (IBA), National Centre for Scientific Research “Demokritos”

## Abstract

The rising number of immunocompromised individuals, combined with the severity of fungal infections in the general population, has contributed to a significant increase in opportunistic fungal infections, which are often associated with high mortality rates. Existing antifungal drugs, although effective, operate via a narrow range of mechanisms, leading to the rapid development of resistance, while they also primarily target growing host cells. Therefore, the need to develop next-generation antifungal agents that function via a broad range of mechanisms and/or to target dormant/quiescent cells is of great importance. In the present study, we investigate the characteristics and the potential antifungal properties of a series of copper-chelated hyperbranched polyethyleneimines (PEI-Cu) of various Cu:primary amino groups of PEI (Cu:N) molar ratios, using the opportunistic pathogen Aspergillus nidulans as a model microorganism. Our results showed that, despite their dissociation, the PEI-Cu¼ and PEI-Cu〿16 complexes with Cu:N molar ratios of 1:4 and 1:16, respectively, exhibit an apparent fungicidal activity on A. nidulans quiescent conidiospores, while in A. nidulans germlings they affect the hyphal growth rate, produce ROS and alter their mitochondrial morphology network. In addition, no cytotoxic effects were observed on normal human skin fibroblasts at concentrations and incubation times that were entirely inhibitory for A. nidulans. Overall, our results suggest that the investigated PEI-Cu complexes are promising antifungals and their underlying mechanism of action deserves further investigation, especially against drug-resistant quiescent fungal cells.

## 1. INTRODUCTION

Over the last years, the rise and severity of fungal infections overall, and especially in immunocompromised patients has become clearly evident. The surge of pathogenic fungal diseases is attributed, among other things, to the recent pandemic, increasing resistance to antifungal agents, the increased number of severely ill patients or even to climate change and increased international travel [1,2]. Recent studies estimate an annual incidence of 6.5 million invasive fungal infections and 3.8 million deaths, with about 2.5 million (68%) directly attributable to fungal diseases [3]. Opportunistic fungal pathogens, such as *Candida* and *Aspergillus* species (mainly *Aspergillus fumigatus*), pose severe threat to human health when they infect mostly immunocompromised patients [4]. In addition, a World Health Organization (WHO) 2024 data call was released aiming to collect data on antifungal agents in the preclinical development, highlighting, among others, the need for novel antifungal agents [5,6]. Existing antifungal drugs, although effective, operate via a narrow range of mechanisms, leading to the rapid development of resistance. Moreover, “persisters”, a subpopulation of dormant slow growing fungal cells, are very tolerant towards the systematic antifungal agents currently in use, since most of them target growing cells [7]. One type of fungal persistent conidia is found in a particular state of the cell cycle, called quiescence. The development of novel potent antifungals with enhanced safety profiles on humans is a need but also a challenge, as human and fungal cells share a number of common biological pathways [8,9]. Current research on novel antifungals primarily focuses on developing agents operating via a broad range of mechanisms. This includes the exploration of small molecules and polymeric compounds of either synthetic or natural origin with reported promising antimicrobial/antibacterial properties [10–13], which also target quiescent fungal cells [13]. The mode of action of the latter, being different from that of currently available small-molecule antifungals, has been attributed to fungal membrane depolarization or membrane permeabilization/rapture, followed by cell entry and subcellular localization that presumably is furthermore interfering with various subcellular functions/biochemical paths [10,12].

*Aspergillus nidulans (A. nidulans)* is a well-established model microorganism for genetics and molecular/cell biology studies, while it is extensively used as a tractable and versatile organism for the study of pathogenic fungi. Although this fungus is an opportunistic pathogen usually harmless to humans, evidence shows that it is a major cause of invasive aspergillosis (IA) in patients with chronic granulomatous disease (CGD) [14]. In addition, primary cutaneous *A. nidulans* infection has been long since reported to occur at sites of skin injury (e.g. at intravenous access catheter sites or at sites associated with burns or surgery), especially in immunocompromised patients [15,16]. Eisosomes, which are lateral compartments of the plasma membrane [17–19], involved in fungal persistence and pathogenicity [20], are induced in *A. nidulans* quiescent conidia and germlings upon exposure to high copper concentrations (Sophianopoulou and Athanasopoulos, unpublished data). This induction suggests a role for eisosomes and copper in cellular responses to metal stress and resilience to antifungals. As a trace element, Cu serves as an essential cofactor of enzymes that function in many biological processes including iron acquisition, antioxidative defense and energy metabolism [21]. However, elevated intracellular Cu concentrations are toxic, as Cu is well-known to promote lipid peroxidation [22]. Additionally Cu is also established as an antimicrobial agent [23], exerting its effects through multiple mechanisms, including the induction of reactive oxygen species (ROS) production [24].Therefore, its accumulation should be tightly monitored and kept at homeostatic levels.

In this context, it should be noted that when Cu concentration becomes limiting, *Saccharomyces cerevisiae* (*S. cerevisiae)* promotes Cu import through the activation of the transcription factor Mac1 that induces the transcription of the high affinity Cu membrane transporters Ctr1 and Ctr3 [25]. In *S. cerevisiae*, as we have recently shown, eisosomes host Flavodoxin-like proteins (FLPs), enzymes essential for the extra-mitochondrial regeneration of the antioxidant ubiquinol which counteracts lipid peroxidation, in parallel with the well-studied system of glutathione (GPX), and thus protect yeast quiescent/persistent cells from ferroptosis [26]. Thus, the role of copper in fungal homeostasis should be considered in the context of the development of new antifungal agents, possibly exploiting copper modulation - for instance, through the use of copper (nano)carriers - as an antifungal strategy.

Among the systematically studied group of polymers as antifungal/antimicrobial agents, dendritic polymers, encompassing dendrimers, dendrons, and hyperbranched polymers, have been extensively investigated for more than fifty years. They are nowadays recognized as an important macromolecular class, primarily due to their chemical architecture, which favors the encapsulation of drugs, and their large number of end-groups that after appropriate functionalization, or multifunctionalization, allow the tailor-made modification of their structure and properties that can have chemical, biochemical and biological significance [27]. An emerging subclass of macromolecules is the so-called metallopolymers, and of particular interest the metallodendrimers, metallodendrons and metallohyperbranched polymers. These compounds are macromolecules containing metals at different sites of the dendritic architecture. The metal centers can be either attached to the repeating unit of the macromolecule covalently, i.e. by an organic linker, or non-covalently, i.e. through metal chelation [28]. In the first case an essentially irreversible binding of the metal is established, while in the latter case, the metal ion is bound to the donor atoms of the chelating repeating units and/or end groups only by coordination bonds [20]. This reversible binding can potentially lead to the release of the metal ion, if the appropriate conditions are met, in stark contrast to the essentially irreversible binding if the bonds of metals with the polymer are of covalent nature [20,28]. Therefore, special attention is given to metal chelates based on hyperbranched polymers due to the reversible nature of this type of binding, but primarily due to their low cost, availability, accessibility and similarity to dendrimers and dendrons [20,29,30].

**Figure 1:**
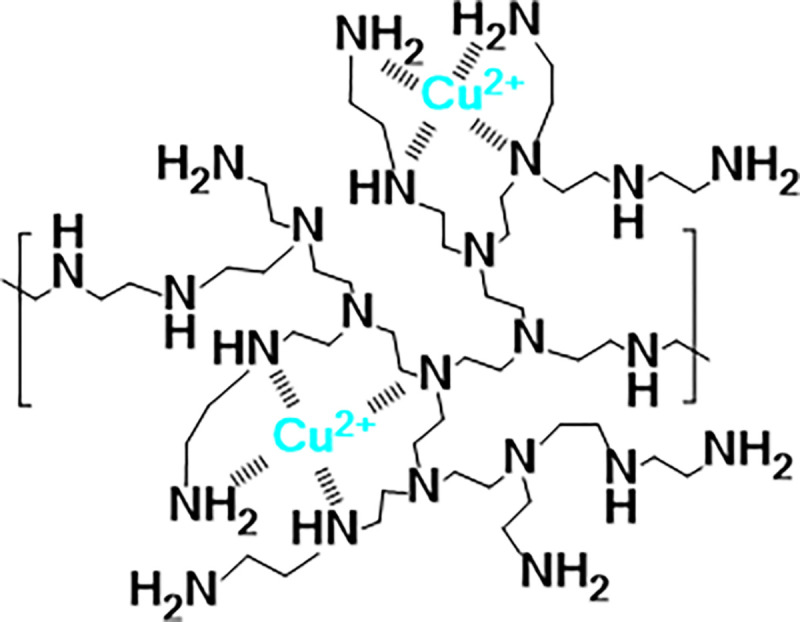
Schematic illustration of possible structures of a polyamine–copper compound with a Cu:primary amino groups molar ratio of 1:4, where Cu is coordinated with both primary, secondary, and tertiary nitrogens.

*N*-donor ligands is one of the most frequently employed routes for chelation of metal ions, while among the hyperbranched polymers, hyperbranched polyethyleneimine (PEI), either as is or appropriately functionalized, is the primary choice [20]. PEI itself has shown antimicrobial properties against bacteria [31–33], yeast [33], other fungi [13,34], and other microorganisms such as algae [35]. Its chemical structure (Fig. 1) favors the formation of strong coordination bonds with labile metal ions particularly with copper(II) ions forming blue-colored water-soluble complexes with stability constant of ca. 10^16^ [18,36,37]. Previous studies of systems based on PEI-Cu complexes, revealed that they are characterized by high water solubility, low cell toxicity, and can stabilize, by coordination, large quantities of Cu(II). Additionally, it has been reported that they can cross the plasma membrane of the yeast*Pichia pastoris*. In this context, a functionalized poly(propylene amine) dendrimer - a compound with a chemical structure similar to that of PEI - carrying Cu(II) ions showed good antimicrobial properties, particularly against Gram-positive bacteria and the yeast *Candida lipolytica* [38], most probably due to its involvement in cell membrane disruption and permeation. The increased permeation can be explained by Tweedy’s chelation theory [39], according to which, the polarity of the metal ion is considerably reduced upon chelation, which enhances the diffusion of such metallodendrimers through the lipid bilayer of cell membranes.

Our recent findings demonstrated the antifungal activity of PEI and its functionalized derivatives against quiescent/persistent conidia and germlings of *A. nidulans* [13]. Given that copper metabolism is linked to the virulence of fungal cells, since its presence has been shown to increase tolerance to certain antifungals [40], in the present study, we focus on the antifungal properties of PEI-copper complexes (PEI-Cu) against fungal quiescent conidia and germlings, in an effort to diminish their pathogenicity by targeting antioxidant mechanisms important for their survival. We developed a series of PEI-Cu complexes of various Cu:N molar ratios (from 1:32 to 1:4) and then we focused on PEI-Cu**¼** and PEI-Cu**⅟**_**16**_ complexes of the highest (1:4) and a medium (1:16) chelated copper molar content, comparing their antifungal activity to that of PEI by monitoring *A. nidulans* growth rate and quiescent conidia survival. The effect of the complexes on normal human skin fibroblasts’ viability was also assessed to rule out the possibility of undesirable cytotoxicity. Finally, the mechanism of action of PEI-Cucomplexes with regard to their antifungal activity was investigated.

## 2. MATERIALS AND METHODS

### 2.1 Chemicals and Reagents

Hyperbranched polyethyleneimine (PEI) of average molecular weight 25 kDa (Lupasol^®^ WF, 99 %, CAS 9002–98-6) was kindly donated by BASF (Ludwigshafen, Germany). Fluorescein isothiocyanate (FITC, CAS 3326–32-7) was purchased from Molecular Probes (Eugene, Oregon, USA). CuCl_2_.2H_2_O (>99.0%, Aldrich, Poole, UK) was used as received.

Dulbecco’s Modified Eagle Medium (DMEM) was purchased from Biosera (Nuaille, France), phenol red-free DMEM and penicillin/streptomycin solution were supplied by Pan Biotech (Aidenbach, Germany), fetal bovine serum (FBS) and trypsin solution were purchased from Gibco (Thermo Fisher Scientific, Waltham, MA, USA). 3-(4,5-dimethylthiazol-2-yl)-2,5-diphenyltetrazolium bromide (MTT) and isopropanol were purchased from Sigma-Aldrich Ltd (Poole, UK). High-purity solvents were obtained from Merck KGaA (Calbiochem^®^, Darmstadt, Germany.

### 2.2 Synthesis and characterization of Fluorescein Isothiocyanate-Labeled (FITC-Labeled) Hyperbranched Polyethyleneimine (PEI-FITC)

The chemical structure of PEI is consisting of ethylenediamine-type branches bearing both primary, secondary and tertiary amine groups (Fig. 1). Inverse gated decoupling ^13^C NMR experiment was employed to determine the primary:secondary:tertiary amine ratio of PEI, its branching degree and its degree of polymerization as described in the literature [41,42]. It was found that the ratio of primary to secondary to tertiary amines of PEI is 1.00:1.18:1.01, the branching degree is 0.68 and the average number of primary amino groups per macromolecule is 183 [13].

Fluorescein isothiocyanate-labeled PEI (PEI-FTIC) was prepared following the interaction of PEI with fluorescein isothiocyanate (FITC) at 10% molar excess in freshly distilled dried dimethylformamide (DMF) in the presence of catalytic amounts of triethylamine, for 24 h at room temperature in the dark, and purified as previously described [13]. The hyperbranched derivative/FITC molar ratio was also determined as previously described and found to be 1:1.1.

### 2.3 PEI-Cu and PEI-FITC-Cu complex formation and characterization

To a PEI solution (2 mg mL^−1^) in phosphate buffer (PB) (pH 7.4, 100 mM) calculated quantities of a CuCl_2_ solution (20 mg mL^−1^ in water) were slowly added under intense stirring yielding to a series of PEI-Cu complexes with various Cu:primary amino groups of PEI (Cu:N) molar ratios, and specifically with 1:3, 1:3.5, 1:4, 1:8, 1:16, 1:32 molar ratios. Although complex formation manifested from the appearance of an intense blue color occurred simultaneously, the resulting solutions were allowed for 24 h at room temperature to ensure that complete equilibrium was reached. In a similar manner, PEI-FITC was employed for the preparation of two PEI-FITC-Cu compounds, notably PEI-FITC-Cu¼ and PEI-FITC-Cu**⅟**_**16**_ having Cu:N molar ratios of 1:4 and 1:16, respectively.

Cu coordination complex formation was then verified by means of UV–Vis spectroscopy, employing a Cary 100 Conc UV–Visible spectrophotometer (Varian Inc., Mulgrave, Victoria, Australia). The corresponding spectra were obtained at the wavelength range of 300–900 nm and the absorption spectra intensity at 632 nm was registered. The apparent hydrodynamic diameter and surface charge of the parent PEI and the PEI-Cu polymerswere determined at 25°C by dynamic light scattering (DLS) and *ζ*-potential measurements, respectively, by employing a Zetasizer Nano ZS apparatus (Malvern Instruments Ltd., Worcestershire, UK). For these experiments, aqueous solutions of PEI or PEI-Cu (10 mg mL^−1^, pH=7.4) were employed. For each solution, ten measurements were acquired, and the results were averaged.

### 2.4 A. nidulans strains, media and growth conditions

Minimal media (MM), complete media (CM) and growth conditions for *A. nidulans* have been described by Cove DJ, 1966. Supplements were added, when necessary, at the adequate concentrations (https://www.fgsc.net/). For growth experiments, conidiospores were inoculated on solid MM or CM supplemented with the appropriate nitrogen source and incubated at 37 °C for 3 – 4 days. The morphological mutations of the *A. nidulans* strains are compiled from A.J. Clutterbuck (https://fungidb.org/fungidb/app). The auxotrophic strain *pabaA1* (p-aminobenzoic acid requiring), originating from the wild-type Glasgow FGSC4 strain (https://fungidb.org/fungidb/app/record/dataset/NCBITAXON_227321), was used as a wild-type strain in this study. In brief, *pabaA1* strain was grown in MM or CM (pH 6.8) supplemented with 5 mM urea (CAS 57–13-6) as sole nitrogen source, 1% (w/v) glucose (CAS 50–99-7) as sole carbon source and 5 mM p-aminobenzoic acid (PABA, CAS Number: 150–13-0). Conidia in CM were harvested and transferred to 10 mL of a 0.05% Tween-20 (CAS 9005–64-5) sterilized aqueous solution. Conidia suspension was filtered through Miracloth (475855–1R EMD Millipore) and after successive decimal dilutions (1:10), their concentration was calculated using a Neubauer hemocytometer.

### 2.5 Minimum Inhibitory Concentration (MIC) determination of PEI-Cu complexes

The MIC of each PEI-Cu complex was determined in 96-well flat bottom plates (Greiner AG, Austria), using a slightly modified EUCAST E.DEF 9.3 method for moulds. Briefly, the assay mixture was prepared by adding 1 volume (0.1 mL) of conidial suspension (~ 2×10^5^ conidia mL^−1^ in 0.05% Tween-20) to 1 volume of assay medium [MM DS (Double Strength)] containing the tested compound in two-fold concentrations. Serial dilutions of each compound were prepared in the assay mixture, with final concentrations ranging from 1 to 20 μg mL^−1^. The plates were sealed with plastic membrane to reduce evaporation and incubated at 37 °C for 48 h. After incubation, the plates were visually analyzed by assessing the degree of hyphal growth. The concentration of each compound yielding no visible hyphae growth was recorded as the MIC value. Six technical replicates were included in the plate for each concentration, and the experiment was repeated at least five independent times.

### 2.6 Colony formation assay

To investigate the toxicity of PEI-Cu complexes, the colony forming unit (CFU) assay was used, in a slightly modified protocol described previously [13]. In brief, the assay mixture was prepared as mentioned in section 2.5 with a final inocula concentration of 1×10^5^ conidia mL^−1^ and 5 or 50 μg mL^−1^ of each compound (i.e. ~MIC and 10 × MIC). The plates were incubated at 37 °C for 2, 4 and 6 h. After incubation, each well content (0.2 mL) was diluted 1:10 in Tween-20 (0.01% v/v). One hundred μL (100 μL) of each suspension was spread into CM plates, yielding an approximate of 300 cells per plate, and the plates were incubated for 2 – 3 days at 37 °C. The number of colonies was then counted for each sample and compared to that of the control sample (no added compounds). Relative cell viability was calculated as the percentage (%) of cell survival relative to the number of colonies formed in the control sample. Three replicates were performed for each compound concentration, and the experiment was independently repeated at least 3 times.

### 2.7 DCF assay for ROS detection

To detect accumulation of reactive oxygen species (ROS), the 2’,7’-dichlorodihydrofluorescein diacetate (H_2_DCFDA) assay was used. H_2_DCFDA is a widely used fluorescent probe for ROS detection that is cell-permeable and non-fluorescent until it is hydrolyzed by intracellular esterases and subsequently oxidized by ROS, to form the highly fluorescent dichlorodihydrofluorescein (DCF). In particular, 35 mm Petri dishes containing 2 mL of MM and the appropriate auxotrophy (p-aminobenzoic acid) were inoculated with ~5 × 10^5^ *pabaA1* conidia mL^−1^, for 14 h at 25 °C. 10 μM of H_2_DCFDA was added to each dish and the dishes were incubated in the dark for at least 45 min, at 25 °C. Their medium was then replaced with 2 mL of MM containing 5 μg mL^−1^ of each test compound, separately, in the absence or presence of 10 mM of the ROS scavenger N-acetyl cysteine (NAC), and the plates were incubated in the dark for either 5 or 25 min. Treated conidia were observed by epifluorescence microscopy and ROS accumulation was quantified by calculating the percentage (%) of cells exhibiting DCF fluorescence compared to the total number of cells. Approximately 150 cells were observed in each sample and the experiment was repeated 3 times (n=450).

### 2.8 Confocal Laser Microscopy (CLM)

*A. nidulans pabaA1* 1×10^6^ conidia mL^−1^ in 0.05% (v/v) Tween-20 were cultured in MM supplemented with 5 mM urea and 1% (w/v) glucose as sole nitrogen and carbon source, respectively, in 8-well μ-Slides (Ibidi GmbH, Germany). Cells were imaged using a Leica TCS SP5 (Leica Microsystems Ltd., Milton Keynes, UK) confocal microscope, with a Leica HCX PL APO CS 63x/1.4 NA oil immersion lens. FITC was excited with a 496 nm laser line. Images were acquired and processed using Fiji ImageJ software [43], as described in Athanasopoulos et al., 2015 [18].

### 2.9 Time-course Confocal Laser Microscopy (CLM) and hyphal growth rate measurements

The effect of each PEI-Cu compound on hyphal growth rate was evaluated using confocal laser microcopy (CLM) images and subsequent image analysis. Conidia were inoculated into sterile ibidi 8-well coverslips. Each well contained 200 μL of MM and ~5 × 10^5^ *pabaA1* conidia mL^−1^. The cells were incubated at 25 °C for 12 – 14 h. Following incubation, the ibidi chambers were transferred to the confocal microscope chamber, where 5 μg mL^−1^of each of the test compound was added, separately, and imaging was started immediately thereafter. Time-course images were captured every 10 min over a 90-min period, with at least twenty individual cells measured per sample. Following image acquisition, hyphal growth rates were calculated using the MTrackJ plugin in Fiji ImageJ software (version 1.54f, National Institutes of Health, Bethesda, MD, USA) by measuring the elongation of individual cells expressed in μm/min [44].

### 2.10 Epifluorescence and phase contrast microscopy

Coverslips containing the attached hyphal germlings of a *pabaA1* strain were imaged using an Axioplan 2 Observer Z1 fluorescence microscope (Carl Zeiss, Jena, Germany), equipped with Plan-Apochromat 40× or 100×/1.40 NA oil immersion objective lens, and appropriate fluorescence light filter sets. Images were captured with a digital camera (Retiga 2000R, QImaging, Surrey, BC, Canada) and Occular version 2.01 acquisition software (Teledyne Photometrics Inc., Tucson, AR, USA) and were processed with Fiji ImageJ software [45].

### 2.11 Cytostatic effect of PEI-Cu complexes

To investigate the cytostatic effect of PEI-Cu complexes against *A. nidulans* quiescent conidia, ~10^6^ mL^−1^ *pabaA1* conidia in 0.05% Tween-20 were cultured in 2 mL of MM supplemented with 5 mM urea, 1% (w/v) glucose and 5 μg mL^−1^of each of the PEI-Cu complexes (PEI-Cu**¼** or PEI-Cu**⅟**_**16**_) tested. Cultures were grown on coverslips in 35 mm Petri dishes, for 12 – 14 h at 25 °C. Cell images were captured using an Axioplan 2 fluorescence microscope as previously described (see 2.10).

### 2.12 Mitochondrial morphology assay

To assess the impact of PEI-Cu complexes on the mitochondrial morphology of *A. nidulans* germlings, ~5 × 10^6^ mL^−1^ *pabaA1* conidia in 0.05% (v/v) Tween-20 were cultured in 2 mL of MM supplemented with 5 mM urea and 1% (w/v) glucose, as sole nitrogen and carbon source, respectively, in 35 mm Petri dishes containing a coverslip, for 12 – 14 h at 25 °C. 5 μg mL^−1^ of PEI, PEI-Cu**¼**or PEI-Cu**⅟**_**16**_ were added and the samples were incubated for an additional 5 and / or 15 min. For mitochondrial staining, 0.5 μM of the fluorescent probe MitoView^™^ 650 (Biotium) was added to each sample at least 15 min prior to imaging, while protecting the samples from light exposure. Following staining, the coverslips with attached hyphal germlings were mounted onto microscope slides and visualized, using an Axionplan 2 fluorescence microscope as previously described (see 2.10). The mitochondrial network of the cells was classified as either tubular or non-tubular (moderate or heavily fragmented), and the percentage of cells displaying a tubular mitochondrial network was measured for each sample. Approximately 200 cells were observed in each sample and the experiment was repeated 3 times (n=600).

### 2.13 Assessment of PEI-Cu complex stability

To assess the stability of PEI-Cu complexes and consequently intracellular Cu availability, a functional assay based on the pigmentation of *A. nidulans* conidia was used [46,47]. This approach is based on the requirement of copper for the activity of laccases, enzymes responsible for melanin synthesis in *A. nidulans* conidia (for more details see results). In particular, *A. nidulans* ~10^6^ mL^−1^ *pabaA1* conidia were inoculated into 96-well microplates containing 0.2 mL of copper-free liquid MM and each of the PEI-Cu polymers, separately. The concentration of each polymer was adjusted to achieve a final copper concentration of 1.6 μM in each sample, equivalent to 0.8 μg mL^−1^ of PEI-Cu at a 1:4 ratio. Cultures were incubated at 37 °C for 90 min, plated on 35 mm Petri dishes containing copper-free MM and allowed to grow for 3 – 4 days at 37 °C. Subsequently, conidial pigmentation was observed.

### 2.14 Human cells, cell culture conditions and cell viability assay

Normal human skin fibroblasts (AG01523) used in this study were obtained from the Coriell Cell Repositories (Camden, NJ, USA). Cells were routinely cultured in DMEM containing penicillin (100 U mL^−1^), streptomycin (100 μg mL^−1^) and 15 % FBS and were regularly tested to ensure their mycoplasma-free status. Cultures were maintained in a humidified atmosphere, at 37 °C and 5 % CO_2_ and cells were subcultured when necessary, using a trypsin/citrate (0.25 %/0.30 %, w/v) solution. The cell viability assay was performed as previously reported [48]. In brief, cells were cultured in 96-well plates until confluence, before the addition of PEI-Cu complexes at concentrations of 5–100 μg mL^−1^ for 4, 12 and 24 h. Putative cytotoxic effects of PEI and of CuCl_2_ corresponding to the nominal copper ion concentration of the Cu: N = 1:4 derivative at the same concentrations were also assessed, while untreated cells served as the control samples. At the end of the incubation periods, culture medium was aspirated, replaced by an MTT solution (1 mg mL^−1^ in phenol red-free DMEM) and cells were further incubated for 4 h. Produced formazan crystals were dissolved in isopropanol, optical density at 550 nm was measured using a Spark microplate analyzer (Tecan, Männedorf, Switzerland) and cell viability was estimated as % ratio of the untreated control. At least three independent experiments conducted in triplicates were performed.

### 2.15 Statistical Analysis

At least three independent repetitions for each experiment were performed, and the data are presented as mean ± standard deviation. A Student’s t-test was employed to assess statistical significance for all treatments (ns, * p < 0.05, ** p < 0.01, *** p < 0.001; ns not significant).

## 3. RESULTS

### 3.1 Formation of PEI–Cu(II) complexes

The coordination of Cu(II) with branched polyethyleneimine, which provides, to the best of our knowledge, the first polyamine-metal complex, was originally described in 1963 [38] and ever since studied by many research groups [20,27–29,31–34,49]. Thus it became evident that branched polyethyleneimines, of different sources, molecular weights and branched microstructures, always form highly water soluble chelates with copper(II) ions, having an intense blue color and exhibiting a maximum in UV-Vis spectroscopy at 632–635 nm, with high stability constants [20,27–29,31–34,49]. However, PEI is a strong base and at concentrations employed throughout our experiments (up to 500 μg mL^−1^ in either minimal media or complete media for fungi or mammalian cells) the pH of the culture solutions was found to increase considerably. It was therefore necessary to employ a phosphate buffer (PB, pH=7.4, 100 mM) as well as to examine the conditions, specifically the Cu:primary amino groups of PEI (Cu:N) molar ratios, that fully ensure complete binding of Cu(II) ions in this buffer, in order to avoid any effects that could be due to the presence of free copper ions in our working solutions. To this end, we followed the formation of PEI-Cu complex using UV–Vis spectroscopy in PB, taking advantage of the strong absorbance of PEI-Cu solutions [both PEI solutions and free Cu(II) solutions at this wavelength and at the employed concentrations have negligible absorption]. Their absorbance spectra follow the Beer–Lambert law, and exhibit a maximum at 632 nm resulting from the $d_{xz/yz}(Cu)\;->\;d_{x2-y2}(Cu)$ charge transfer [50]. Consequently, complex formation in PB was followed by monitoring the absorbance of a PEI solution upon addition of increasing quantities of Cu(II). When PEI is in excess, the absorbance is expected to be a linear function of the Cu:N ratio, denoting quantitative formation of the chelate, while when the absorbance deviates from the straight line it signifies incomplete complex formation and the presence of uncomplexed (free) Cu(II) ions [51,52]. As shown in Fig. S1A (Supporting Information), the absorbance maximum of all PEI-Cu complexes is located at 632 nm, while by plotting the absorbance maximum vs. Cu:N molar ratios (see Supplementary Fig. S1B) the observed linearity indicates quantitative complex formation up to 1:4 Cu:N molar ratio, which was, thereafter, the highest molar ratio employed in this study. Accordingly, PEI was not loaded with the maximum stoichiometric amount of Cu(II) ions as usually employed in previous publications that study these complexes up to a Cu:total number of PEI nitrogen groups equal to 4 or even to 5 (CuN4 and CuN5) due to the expected four-coordination or even to five-coordination complexes [38–40,53,54]. Instead, we decided to work with solutions having excess of the ligand, in order to ensure complete binding of Cu(II) ions. The registered absorbance maximum at 632 nm is pointing to four atoms of N attached to each atom of Cu(II) and to a planar array of the four Cu-N bonds (Fig. 1) [53–56]. PEI due to its branched structure that contains both primary, secondary, and tertiary nitrogen atoms can be regarded not only as a homologue of the linear ethylenediamine or triethylenetetramine [54], but also as derivatives of branched oligoamines, such as tris(2-aminoethyl)amine(tren) [53], all of which are known to form strong complexes with Cu of planar geometry [32,33,51,52,57]. It is, therefore, reasonable to assume that Cu is coordinated with both primary, secondary, and tertiary nitrogens of PEI, and this can also be the reason of additional complex stability.

Dynamic light scattering experiments reveal a moderate increase of polymer hydrodynamic size upon addition of Cu ions up to a Cu:N ratio of 1:16, which is also accompanied by a concomitant increase in zeta potential (see Supplementary Fig. S2). Upon further increasing the Cu:N ratio the zeta-potential remains constant, while the size of the polymer is slightly reduced although this size reduction is not statistically significant. The observed size and zeta-potential increase is expected given that copper ions complexation within the hyperbranched scaffold results in an increase of its overall positive charge. This is manifested not only in the observed charge density increase but also in the size increase of the polymeric nanoparticle due to increased electrostatic repulsions between the positively charged moieties. The observed slight decrease in size upon further addition of Cu ions most probably is the result of a large number of Cu complexes formed that cause ethyleneimine branches to approach each other (Fig. 1) affording more compact nanoparticles.

### 3.2 Growth inhibition of quiescent fungal cells by PEI–Cu(II) complexes

**Figure 2:**
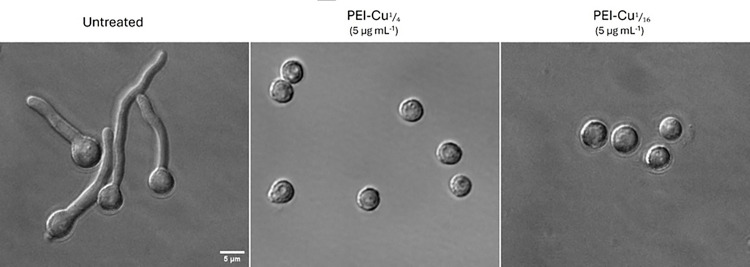
Growth inhibitory effects of PEI-Cu¼ and PEI-Cu complexes against quiescent fungal cells of A. nidulans. Representative phase contrast microscopy images of pabaA1 conidiospores grown for 14 h in the presence of 5 μg mL^−1^ PEI-Cu¼ or PEI-Cu at 25 °C.

The growth inhibitory effects of PEI-Cu**¼** and PEI-Cu**⅟**_**16**_ on quiescent conidiospores of *A. nidulans* wild-type strain were investigated. Conidiospores in MM were treated with PEI-Cu**¼** and PEI-Cu**⅟**_**16**_, at near-MIC concentrations (see Table 1), for 14 h at 25 °C and 48 h at 37 °C, and observed visually for their ability to germinate and form mycelia compared to untreated conidiospores. The results presented in Fig. 2 show that in the presence of the test PEI-Cu complexes, fungal conidiospores do not germinate to form hyphae, in accordance to our previously reported data using PEI [13]. More specifically, treated conidiospores remain at their isotropic growth phase compared to their untreated counterparts, which proceed to the stage of establishment and maintenance of polarity, and eventually generate the observed hyphae. These results suggest that PEI-Cu**¼** and PEI-Cu**⅟**_**16**_ complexes exhibit fungistatic properties against quiescent *A. nidulans* conidiospores.

The MIC values for the PEI-Cu complexes were estimated to be 7.5 μg mL^−1^ for PEI-Cu**⅟**_**16**_ and 4 μg mL^−1^ for PEI-Cu**¼**, in the same range as that of PEI, as shown in Table 1.

### 3.3 Effect of PEI–Cu(II) complexes on normal human skin fibroblasts’ viability

**Figure 3:**
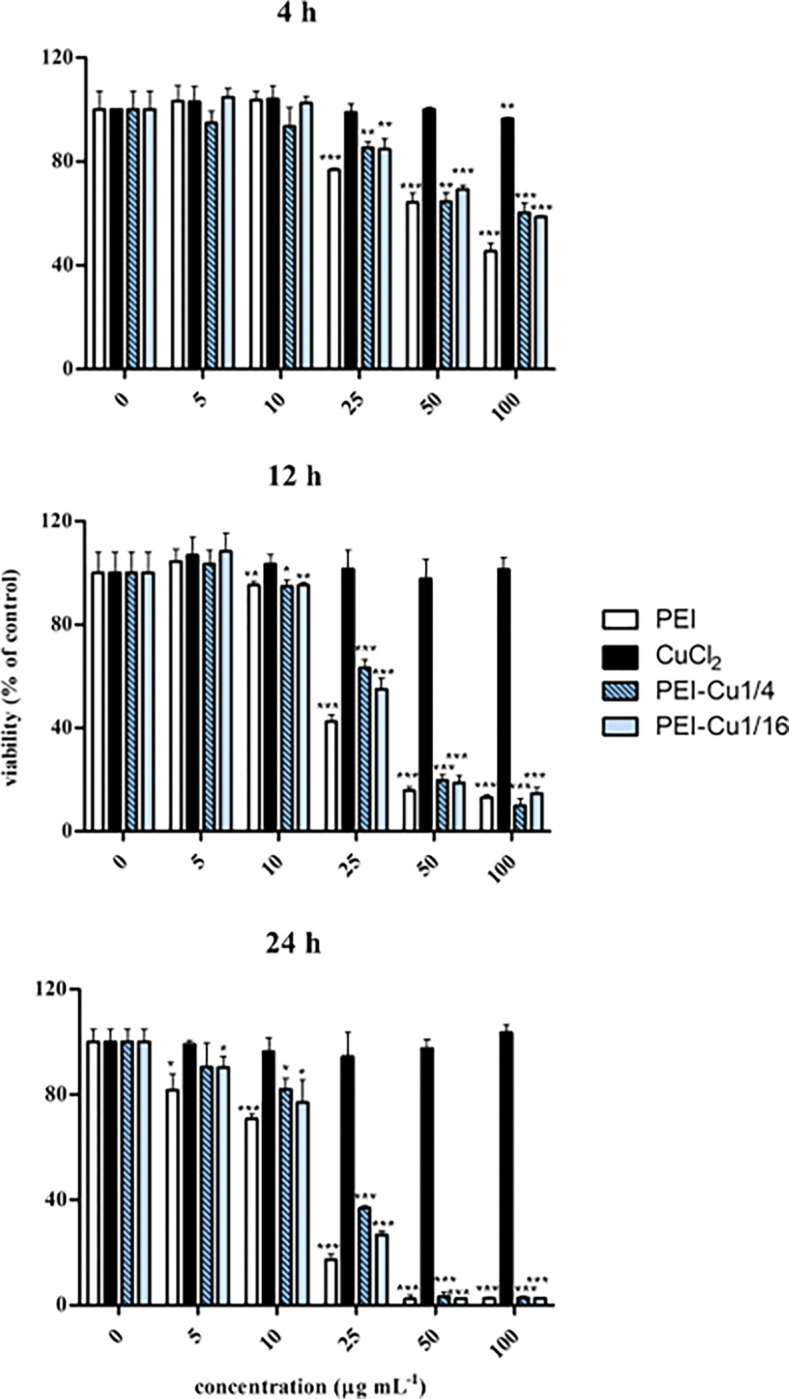
Effect of PEI-Cu (II) complexes on the viability of normal human skin fibroblasts, as estimated by the MTT assay. AG01523 cells were plated in 96-well plates until confluence and PEI, PEI-Cu complexes and CuCl_2_ corresponding to the nominal copper ion concentration of the Cu:N=1:4 derivative were added at concentrations from 0 (untreated control) to 100 μg mL^−1^ for 4, 12 and 24 h. At the end of the incubation periods, MTT was added at a concentration of 1 mg mL^−1^ for 4 h and optical density of the solubilized in isopropanol formazan crystals was measured at 550 nm. Cell viability was calculated as a percent ratio of the untreated control samples. Data presented are mean values ± standard deviations. At least three independent experiments were conducted in triplicates with similar results. One representative experiment is depicted here. Asterisks denote statistically significant differences in comparison to the respective control sample.

In order to exclude the possibility of undesirable cytotoxic effects of PEI-Cu complexes on normal human cells, we performed a cell viability assay in cells incubated with various concentrations of the complexes for 4, 12 and 24 h. Experiments were performed in a commercially available line of human skin fibroblasts, given the reported association of *A. nidulans* with cutaneous aspergillosis, especially in immunocompromised patients. As estimated by the MTT assay and shown in Fig. 3, a dose- and time-dependent cytotoxic effect induced by PEI-Cu complexes was observed. However, up to the concentration of 10 μg mL^−1^ of PEI-Cu**¼** and PEI-Cu (which exceeds the MICs calculated for *A. nidulans*), human skin fibroblasts were shown to remain 100 % viable and >90 % viable after a 4- and 12-h incubation, respectively. Even after a 24-h incubation, cells preserved an approx. 90 and 80 % viability at 5 and 10 μg mL^−1^, respectively of PEI-Cu**¼** and PEI-Cu. It should be noted that PEI was found to be slightly more cytotoxic than PEI-Cu complexes, whereas CuCl_2_ was always non cytotoxic for normal human skin fibroblasts. Similar results were obtained when experiments were repeated using primary normal skin fibroblasts (data not shown).

### 3.4 Stability of PEI–Cu(II) complexes

To assess the stability of PEI-Cu complexes, conidial pigmentation was used as an indicator of intracellular copper abundance (see Materials and Methods). Briefly, in the absence of copper, defects in the dark green normal pigmentation of wild-type conidia result most probably from the absence of a functional copper-dependent laccase encoded by the *yA*^*+*^ gene. This enzyme accumulates in the walls of conidia and converts a yellow precursor, an intermediate in the melanin biosynthesis pathway of *A. nidulans*, into melanin, resulting in dark green pigmentation [58]. During intracellular copper deficiency, laccase activity is impaired, as its polymerization is reduced, leading to the accumulation of a yellow intermediate [46]. Overall, conidial pigmentation serves as an indicator of intracellular copper availability, where dark green pigmentation indicates sufficient copper, while yellow pigmentation indicates copper limitation, without affecting the growth rate of *A. nidulans* conidiospores.

**Figure 4:**
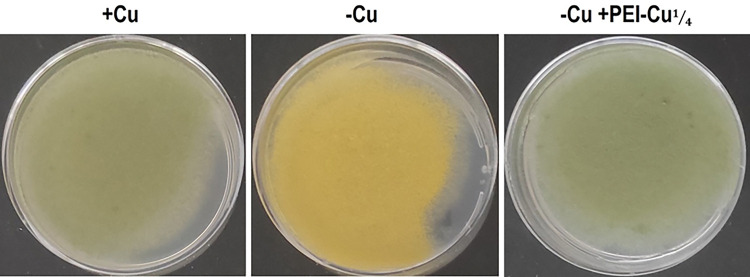
*Growth of A. nidulans conidiospores in copper-supplemented (+Cu) and copper-free (−Cu) minimal media. In the presence of copper, A. nidulans pabaA1 conidia show normal dark green pigmentation (left). In the absence of copper (−Cu), conidia show yellow pigmentation due to reduced polymerization of the copper-dependent laccases (middle). When 0.8 μg mL*^*−1*^ *of PEI-Cu is added in Cu-free (−Cu) media, conidia show again their normal dark green pigmentation (right). Similar results were observed with PEI-Cu* (data not shown).

When either PEI-Cu**¼** or PEI-Cu was added to the copper-free growth MM, the natural green pigmentation was restored in *A. nidulans* conidia (Fig. 4). This result suggests that both PEI-Cu complexes dissociate (outside or/and inside the cell), releasing bioavailable copper ions that diffuse into the cytoplasm at concentrations sufficient to support copper-dependent laccase function, thus restoring normal dark green pigmentation. Despite the differences in copper content between PEI-Cu**¼** and PEI-Cu complexes, the sub-MIC concentration of 0.8 μg mL^−1^ was sufficient to initiate melanin production. This observation confirms that copper is efficiently released from both complexes, rendering it accessible to cellular utilization, particularly under conditions of copper deficiency.

### 3.5 PEI–Cu(II) complexes’ effects on hyphal growth rate of A. nidulans germlings

**Figure 5:**
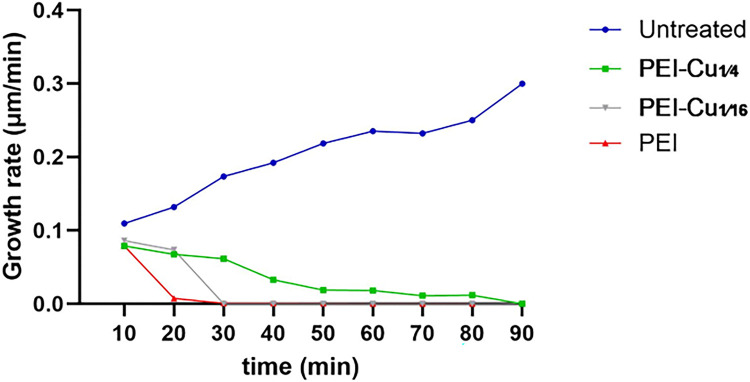
Growth rate of A. nidulans 14 h germlings treated with 5 μg mL^−1^ PEI, PEI-Cu¼, or PEI-Cu at the indicated time points, compared to untreated cells. Germlings were treated with PEI complexes for 90 min at 25 °C and hyphal growth rate (extension) was monitored using live microscopy and an open-source software [44]. Dots represent the average hyphal extension velocity, calculated as the ratio of the distance the hyphal apical tip of the same germling moves between two consecutive microscopy frames (in μm) to the time interval between the frames (in min). All polymers inhibited hyphal growth rate, with PEI and PEI-Cu exhibiting their cytostatic effect within 20 – 30 min, while PEI-Cu¼ within 50–90 min. Data are expressed as a set of at least 30 independent hyphal extension velocity values (μm/min) obtained from at least 3 independent experiments.

Growth rate experiments of *A. nidulans* germlings were performed as described in Athanasopoulos *et al.,* 2021 [44]. Our results revealed that PEI, PEI-Cu**¼**, and PEI-Cu significantly inhibited germlings’ growth rate, as evidenced by the gradual decrease in hyphae extension rate. Both PEI and PEI-Cu exhibited a rapid cytostatic effect, stopping hyphal extension within 20 – 30 min of exposure. In contrast, PEI-Cu**¼** showed a delayed effect, requiring up to 90 min to completely stop hyphal extension, although a significant reduction in the overall growth rate, compared to untreated cells, was already observed from the first time-point examined (10 min) (Fig. 5).

**Figure 6:**
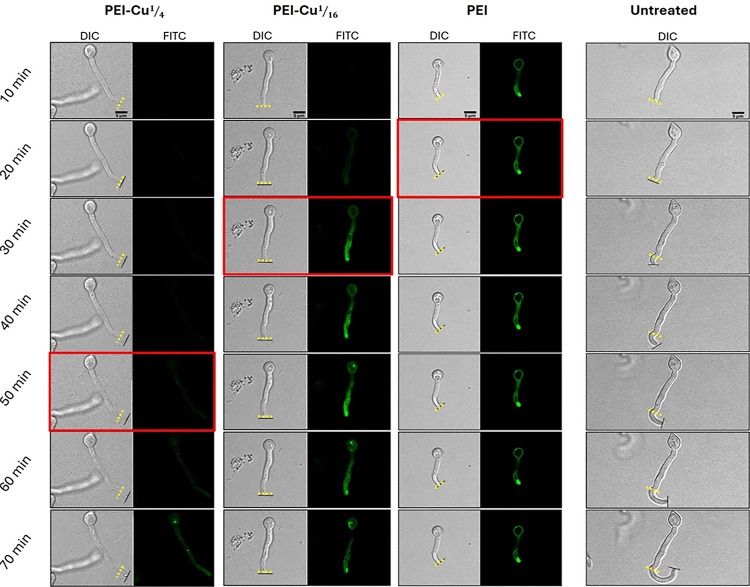
Representative confocal microscopy images illustrating the time course of hyphal growth rate in A. nidulans germlings treated with 5 μg ml^−1^ of PEI-FITC, PEI-FITC-Cu¼ or PEI-FITC-Cu〿_16_ compared to untreated cells. Brightfield images (DIC) show hyphal extension over time, while the fluorescence channel {FITC) shows the localization of the fluorescein-labeled compounds. Yellow dotted lines indicate the initial position of the hyphal tip at the 10 min time point, while black lines represent the current position of the hyphal tip at each subsequent time point. Frames outlined in red represent the time point that the hyphal extension has stopped, indicating growth inhibition. Hyphal extension of the depicted cells was stopped at 20 min of incubation with PEI, 30 min with PEI-FITC-Cu〿_16_ and 50 min with PEI-Cu¼, while growth continued in untreated cells. Accumulation of the test compounds, indicated by increased intracellular FITC-fluorescence, coincides with the onset of the inhibitory effect within a time window of approximately 10 min. The growth inhibition shown in the images reflects individual cell behavior, thus it may differ from the results for average extension velocity, as calculated from multiple hyphae measurements shown in Fig. 5. Scale bar: 5 μm.

As further shown in Fig. 6, temporal differences in inhibition dynamics demonstrate the effect of copper on the antifungal activity of the test PEI polymers. The delayed cytostatic effect of both PEI-Cu**¼** and PEI-Cu, compared to PEI alone, suggests that copper incorporation modulates the antifungal activity of PEI, and more precisely suggests that elevated copper intracellular levels increase the resilience of fungal cells against the fungistatic activity of PEI.

### 3.6 PEI–Cu(II) complexes produce ROS in A. nidulans germinated conidia

**Figure 7:**
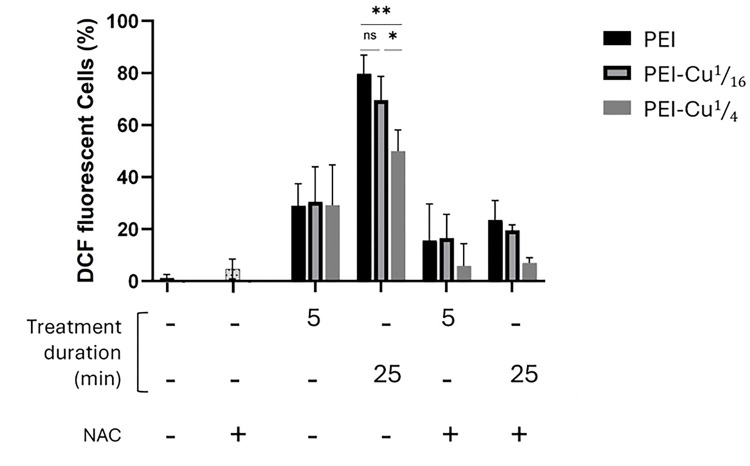
Percentage of cells showing DCF fluorescence compared to the total number of cells observed, under the indicated different experimental conditions. A. nidulans germlings of 14 h growth at 25 °C were incubated for 5 or 25 min in the presence of 5 μg mL^−1^ PEI or PEI-Cu and PEI-Cu¼ complexes. The number of DCF-fluorescent cells was reduced in the presence of the ROS scavenger N-acetyl cysteine (NAC). Data are expressed as mean ± SD, of 3 independent values obtained from at least 3 independent experiments. Total number of cells observed for each sample was 450 (n=450). Asterisks denote statistically significant differences between indicated data sets.

To investigate the role of oxidative stress in the antifungal mechanism of PEI and PEI-Cu complexes, the accumulation of reactive oxygen species (ROS) in *A. nidulans* germinated conidia was evaluated. Using 2’,7’-dichlorodihydrofluorescein diacetate (H_2_DCFDA) in a fluorometric approach to detect ROS production, our results, shown in Fig. 7 revealed that PEI, PEI-Cu, and PEI-Cu**¼** significantly induce ROS production shortly after their addition, with significant DCF fluorescence detection within 5 min of incubation. DCF fluorescence signal increased significantly 25 min post-exposure of the cells to PEI and PEI-Cu complexes (Fig. 7). The rapid accumulation of ROS at 5 min preceded the cytostatic effects of the test compounds observed approximately 15 – 20 min post-incubation of the cells at 25 °C (see Fig. 5, 6), suggesting that oxidative stress could be one of the mechanisms underlying the antifungal activity of the compounds. Notably, 25 min post-exposure, PEI-Cu**¼** showed a lower ROS signal than PEI and PEI-Cu, a result compatible with our data showing that in the presence of copper, the inhibitory effect of PEI on the fungal growth rate was significantly delayed (Fig. 5, 6). In the presence of the ROS scavenger *N*-acetyl cysteine (NAC), the DCF-fluorescent signal was reduced under all conditions tested.

### 3.7 Effects of PEI–Cu(II) complexes on mitochondrial morphology

To further investigate the involvement of oxidative stress in the mechanism underlying antifungal activity of PEI and PEI-Cu complexes, we examined mitochondrial network morphology - as an indicator of the oxidative state of a cell - in *A. nidulans* germlings in the presence of each of the test compounds.

**Figure 8:**
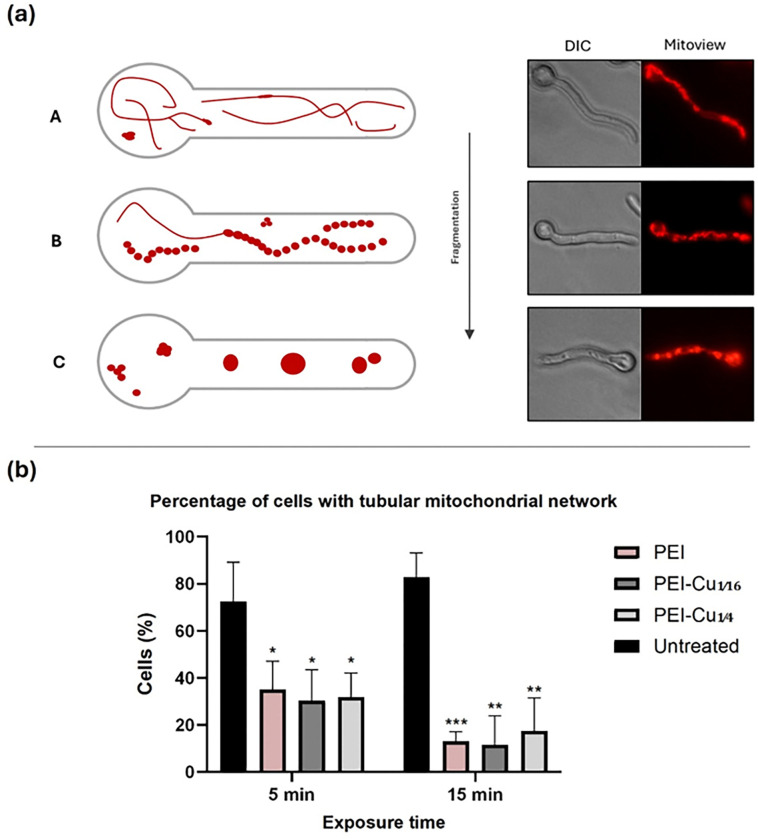
Effects of PEI and PEI-Cu complexes on mitochondrial morphology of A. nidulans. a) Schematic presentation and representative epifluorescence microscopy images of the distribution of mitochondrial network in A. nidulans 14 h germlings, visualized using Mitoview labeling: (A) normal, filamentous/tubular mitochondrial network characterized by continuous, elongated mitochondrial structures forming interconnected tubules; (B) moderate fragmented network displaying a disrupted filamentous morphology with predominantly intermittent discontinuities (doted lines); and (C) heavily fragmented network marked by large, distinct mitochondrial aggregates. These morphological variations reflect progressive changes in mitochondrial integrity. Tubular mitochondria indicate an intact mitochondrial network, indicative of low oxidative stress and normal cellular function [69]. b) Percentage of Mitoview-labeled cells displaying tubular mitochondria following 5- and 15-min exposure to 5 μg mL^−1^ PEI, PEI-Cu¼ or PEI-Cu¼ at 25 °C. Data are expressed as mean ± SD of 3 replicates obtained from at least 3 independent experiments. Asterisks denote statistically significant differences in comparison to the respective control sample.

To approach this, *pabaA1* germlings of 14 h growth were exposed to 5 μg mL^−1^ of PEI, PEI-Cu or PEI-Cu**¼** for 5 and 15 min, subjected to MitoView-labeling, and observed under a fluorescence microscope. Schematic presentation and representative epifluorescence microscopy images of the distribution of mitochondrial network in *A. nidulans* 14 h germlings, visualized using Mitoview labeling is shown in Fig. 8a (for details see legend of Fig. 8). Analysis of epifluorescence microscopy images is presented as the percentage (%) of cells showing tubular mitochondria network (Fig. 8b) in comparison to cells with vesicular mitochondria. Furthermore, the results in Fig. 8b suggest a time-dependent increase in mitochondria network fragmentation, indicative of increased oxidation stress. These early changes in mitochondrial network morphology, which precede the observed apical growth rate inhibitory effects of the test compounds (Fig. 5, 6), suggest that mitochondria function plays a role in the mechanism underlying the function of these compounds, possibly mediated by oxidative stress pathways.

### 3.8 Cytotoxicity studies of PEI–Cu(II) complexes against quiescent fungal cells

**Figure 9:**
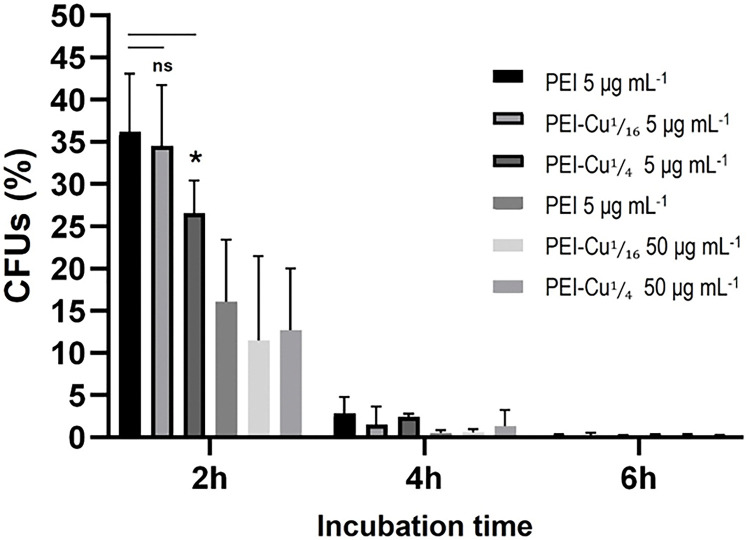
Viability of A. nidulans pabaA1 conidia, expressed as the percentage of colony-forming units (CFU), following treatment with 5 or 50 μg mL^−1^ (MIC and 10x MIC) of PEI and PEI-Cu complexes, at time points of 2, 4, or 6 h. Colony counts were in each case compared to untreated conidia (control), which was defined as 100% of growth. Thus, relative viability was calculated as the percentage of survival of treated conidia compared to the untreated control. Data are expressed as mean ± SD of 6 replicates obtained from at least 3 independent experiments. Asterisks denote statistically significant differences between indicated data sets.

As quiescent fungal conidia are resistant to the available antifungals currently used [26], the cytotoxic activity of PEI-Cu complexes against *A. nidulans* quiescent conidia was evaluated. Based on our previously published data [17], a 2 h exposure period, starting immediately after conidia inoculation to the growth medium, was chosen to target conidiospores in the quiescence state of their cell cycle [59]. A reduction in growth upon treatment with different PEI complexes was observed, calculated relative to the untreated conidia (control, defined as 100% of growth). Conidia survival was significantly reduced to 27% of Colony-Forming Units (CFUs) after treatment with PEI-Cu**¼**, compared to 36% observed after treatment with PEI (Fig. 9). On the other hand, while PEI-Cu also significantly reduced quiescent conidia survival (34%), this reduction was not significantly different from the one observed in PEI-treated conidia (Fig. 9).

A tenfold increase in the concentration of PEI-Cu**⅟**_**16**_ and PEI-Cu**¼** (50 μg mL^−1^) further reduced conidia survival to approximately 12% and 13%, respectively, highlighting a dose-dependent effect. Moreover, extending the incubation time to 4 h and 6 h, further enhanced the cytotoxic effects of all test compounds at MIC and 10x MIC concentrations, emphasizing their time- and dose- dependent cytotoxicity on quiescent fungal cells (Fig. 9).

## DISCUSSION

Aiming to guide research and development and in order to increase public health awareness WHO released in 2022 the “WHO fungal priority pathogens list” [5], which enlists 19 different fungal pathogens divided into critical-, high-, and medium-priority groups. In the so-called critical group of WHO, *Cryptococcus neoformans*, *Candida auris*, *Aspergillus fumigatus* and *Candida albicans* are included. This division is mainly based on their resistance to currently available antifungals. Existing antifungal drugs, although effective, target mainly growing cells and operate via a narrow range of mechanisms, leading to rapid development of resistance. Based on the aforementioned knowledge, it becomes evident that the development of novel antifungal agents with enhanced safety profiles is of priority for public health.

Our recent research showed that PEI has antifungal properties against *A. nidulans* quiescent conidia [13]. Furthermore, different cellular processes such as stress responses, host invasion, commensalism, and antifungal sensitivity were shown to be modulated by Cu availability, as evidenced by the global gene expression dynamic under both copper depletion and excess using RNA-seq in the pathogenic fungus *C. albicans* [40]. Importantly, Cu hypersensitivity has been reported for *C. albicans* mutants lacking the eisosome-resident protein Sur7, although the underlying molecular mechanism still remains elusive [60]. Given the antifungal properties of PEI and the relationship between metabolism and the antifungal sensibility of the transition element copper, we chemically synthesized PEI copper-chelates, to examine their effect(s) on antifungal properties of PEI.

Our results show that both PEI-Cu**¼** and PEI-Cu**⅟**_**16**_ complexes exhibit fungistatic and fungicidal properties (Fig. 2, 9). Importantly, no cytotoxic effects were observed on normal human skin fibroblasts at PEI and PEI-Cu complexes’ concentrations up to 10 μg mL^−1^ and incubation times up to 12 h (Fig. 3) that were found to be entirely inhibitory for *A. nidulans*. It should be noted that normal human skin fibroblasts used in the current study represent an appropriate physiologically relevant *in vitro* cell model, since *A. nidulans* has been implicated in cutaneous infections [15,16]. Lower survival rates of normal human skin fibroblasts were evident only by extending the range of PEI and PEI-Cu complexes’ concentrations and/or incubation times, with concentrations ≥25 μg mL^−1^ (approx. 5-fold the MICs for *A. nidulans*) being considerably cytotoxic, especially in the progress of time (Fig. 3). A tendency of PEI-Cu**¼** and PEI-Cu**⅟**_**16**_ complexes to be less cytotoxic than parent PEI though was shown here. As CuCl_2_ was not found to be cytotoxic even at the highest concentration and incubation time applied, toxic effects on skin fibroblasts - when observed - may rather be attributed to PEI than copper, in accordance to the previously reported cytotoxic effect of PEI when used as transfection reagent in various cell types [61]. Besides, copper has been reported to be beneficial for the skin, participating in the synthesis and stabilization of extracellular matrix proteins and in angiogenesis [62].

Regarding the mode of antifungal activity of PEI and PEI-Cu complexes, comparisons between PEI and its copper complexes show that the higher the intracellular copper concentration, the lower the sensitivity of germlings to antifungals. Specifically, as demonstrated by the growth rate assay (Fig. 5, 6), PEI-Cu**¼** (complex with a higher copper content), exhibited delayed antifungal activity compared to PEI-Cu**⅟**_**16**_, suggesting that coordinated copper delays antifungal activity. The protective effect of copper on *A. nidulans* germlings is consistent with the corresponding reported data for *C. albicans* [40]. The delayed appearance of PEI-Cu**¼** and PEI-Cu in fungal cells, evidenced by their intracellular fluorescence compared to PEI (Fig. 6), suggest that copper modulates PEI internalization. However, once inside the cell, the inhibitory activity of all compounds appears within approximately 10 min (Fig. 5, 6). This suggests that copper could affect cell membrane fluidity or permeability, the internalization sites and/or the kinetics of PEI uptake mechanism(s), rather than its intracellular function. The effect of PEI on the uptake mechanism could be associated with stress responses, as cells may actively alter their internalization pathways in response to metal exposure [63] or the bioactivity of PEI terminal groups could be reduced due to the copper chelation, as reported for other functionalized derivatives [34]. Furthermore, our results show that the copper chelate enhances the fungicidal activity of PEI against quiescent conidia, as demonstrated by the conidia cytotoxicity assay (Fig. 9).

The differences in Reactive Oxygen Species (ROS) production of *A. nidulans* germlings treated with PEI-Cu**¼** and PEI-Cu compared to parent PEI (Fig. 7), suggest that abundance of intracellular copper might protect fungal cells against oxidative stress. Despite the inherent property of copper itself to induce ROS, intracellular ROS levels were significantly lower in hyphae treated with PEI-Cu**¼** than with PEI [64,65]. Significant levels of ROS were detected shortly after exposure to PEI or PEI-Cu complexes, preceding the cytostatic effect, suggesting that oxidative stress is perhaps the cause of cellular death. ROS production within only 5 min is consistent with significant mitochondrial fragmentation, characterized by the transition from tubular to fragmented mitochondrial network in *A. nidulans* germlings, further highlighting the importance of oxidative stress in the antifungal mechanisms of these compounds. Notably, both mitochondrial fragmentation and ROS accumulation are time-dependent, indicating a progressive and cumulative effect. Since oxidative stress pathways directly or indirectly affect mitochondrial function and vice versa [66–68], a link emerges between ROS production, mitochondrial dysfunction, and the antifungal activity of PEI and PEI-Cu complexes.

Further studies are needed to elucidate the molecular mechanisms underlying the antifungal activity of PEI and PEI-Cu complexes. In addition, a rational therapeutic scheme would be required in order to exploit PEI- and PEI-Cu complexes’ antifungal activity on one hand and exclude putative undesirable side effects on infected human skin on the other, given that increasing concentrations and/or incubation times could result in cytotoxic effects also on normal human skin fibroblasts. Towards this direction, a periodical, short-term topical application on cutaneous surfaces could be suggested, e.g. by the incorporation of PEI and PEI-Cu complexes in appropriate cream formulations. Furthermore, the study of new natural and/or synthetic putative antifungal metallopolymers could provide the basis for the development of more antifungal agents that target both dormant and growing fungal cells, with diverse mechanisms of action and therefore more resilient to the development of fungal resistance.

## Figures and Tables

**Table 1.** The minimum inhibitory concentration (MIC) of PEI-Cu¼ and PEI-Cu**⅟**_**16**_ complexes

Compound	MIC (μg mL^−1^)
PEI-Cu**¼**	5±1
PEI-Cu**⅟**_**16**_	6.5±1
PEI	6.5±1

## Data Availability

All data generated or analyzed during this study are included in this published article. All unique/stable reagents generated in this study are available.
